# Xanthan Gum Removal for ^1^H-NMR Analysis of the Intracellular Metabolome of the Bacteria *Xanthomonas axonopodis* pv. *citri 306*

**DOI:** 10.3390/metabo4020218

**Published:** 2014-04-22

**Authors:** Vanessa R. Pegos, Rafael R. Canevarolo, Aline P. Sampaio, Andrea Balan, Ana C. M. Zeri

**Affiliations:** 1 Brazilian Biosciences National Laboratory, Rua Giuseppe Máximo Scolfaro, 10.000-Polo II de Alta Tecnologia, Caixa Postal 6192, CEP 13083-970, Campinas/SP, Brazil; E-Mails: vanessa.pegos@lnbio.cnpem.br (V.R.P.); rafael.canevarolo@lnbio.cnpem.br (R.R.C.); aline.sampaio@lnbio.cnpem.br (A.P.S.); andrea.balan@lnbio.cnpem.br (A.B.); ana.zeri@lnbio.cnpem.br (A.C.M.Z.); 2 Molecular Biology Laboratory, Boldrini Children Hospital, Rua Dr. Gabriel Porto, 1270 Cidade Universitária, CEP 13083-210, Campinas/SP, Brazil

**Keywords:** *Xanthomonas*, xanthan gum removal, ^1^H-NMR, cell extraction protocol, metabolomics of bacteria, metabolomics of microorganisms

## Abstract

*Xanthomonas* is a genus of phytopathogenic bacteria, which produces a slimy, polysaccharide matrix known as xanthan gum, which involves, protects and helps the bacteria during host colonization. Although broadly used as a stabilizer and thickener in the cosmetic and food industries, xanthan gum can be a troubling artifact in molecular investigations due to its rheological properties. In particular, a cross-reaction between reference compounds and the xanthan gum could compromise metabolic quantification by NMR spectroscopy. Aiming at an efficient gum extraction protocol, for a ^1^H-NMR-based metabolic profiling study of *Xanthomonas*, we tested four different interventions on the broadly used methanol-chloroform extraction protocol for the intracellular metabolic contents observation. Lower limits for bacterial pellet volumes for extraction were also probed, and a strategy is illustrated with an initial analysis of *X. citri’s* metabolism by ^1^H-NMR spectroscopy.

## 1. Introduction

*Xanthomonas* is a genus of Gram-negative, yellow-pigmented bacteria which resides at the base of the Gammaproteobacteria and comprises 27 species. Collectively, the genus cause serious diseases in ~400 plant hosts, including a wide variety of economically important crops, such as rice, citrus, banana, cabbage, tomato, pepper and beans [1]. One of its species, *Xanthomonas axonopodis* pv. *citri* (*X. citri*), parasitizes lime, lemon and orange trees’ leaves, stems and fruits, provoking defoliation, blemished fruit, premature fruit drop, die-back of twigs and general debilitation of the tree, thus inflicting considerable economic impact on the citriculture in many subtropical to tropical regions of the world [2].

The genus *Xanthomonas* secretes a peculiar water-soluble, heteropolyssacharidic extracellular matrix named xanthan gum, believed to help the microorganism against diverse environmental constraints during its process of host infestation such as biofilm formation. Mutations in genes related to xanthan gum biosynthesis considerably reduce *Xanthomonas* virulence and, thus, citrus canker symptoms [3]. Due to its rheological properties such as high viscosity and pseudoelasticity, xanthan gum has been widely employed in industry, from a thickening agent of salad dressings and a sausage emulsifier, to a versatile stabilizer of cosmetic products and even some usages in oil drilling and in building products to optimize material properties [4]. Even though it is a desired product in the industry, xanthan gum presence can be a real challenge for some analyses.

A search for studies regarding microorganisms’ metabolomics reveals a crescent but still small number of reports [5,6,7,8,9,10,11], with just a few of them exploiting sample preparation methods [12,13]. Surprisingly, we could find no references regarding adjustments in methodology and protocols directed to the improvement of NMR-based investigations of bacterial metabolome. Although Sana *et al.* [14] studied the differential metabolic response of susceptible or resistant rice plants after a *X. oryzae* infection, to our knowledge no metabolomic studies have dealt specifically with the metabolome of any of the *Xanthomonas*’ species.

The main reason that impelled our efforts in this study rose after an exploratory ^1^H-NMR analysis of the intracellular metabolic content of *X. citri*, extracted by the methanol-chloroform (M/C) method [15] without any specific adaptations. Prior the analysis in the NMR spectrometer, the sample’s viscosity and turbidity already called our attention, indicating that molecules other than metabolites remained in the sample. The NMR spectra acquisition was hampered by the difficulty in setup, specifically with the shimming procedure; furthermore, the signal for the internal reference of concentration (3-(Trimethylsilyl)propanoic acid, TSP) exhibited a considerable reduction in amplitude, suggesting that a cross reaction might be happening between TSP molecules and the interfering substance. We hypothesized that the interfering materials in question were the polysaccharidic constituents of the xanthan gum, once proteins, lipids and genetic material were depleted during the M/C extraction step. The objective of this study was to investigate the effectiveness of four methodological interventions for xanthan gum removal during sample preparation for a NMR spectroscopy analysis of the metabolic content of *X. citri*. Interventions to the intracellular metabolome extraction protocol were added prior to or after the cell lysis step (performed by sonication in a M/C solution).

*In vitro* production of bacteria can be really challenging under abnormal culture conditions (for example, under intentional physicochemical constraints or nutrition restriction), so we turned our attention towards finding out the minimal volume of a pellet of bacteria needed for a robust ^1^H-NMR analysis of the *X. citri* metabolome, expecting that this might be useful to help researchers save their efforts and resources basing their experiments on an optimized protocol.

## 2. Results and Discussion

During a ^1^H-NMR exploratory study of the intracellular metabolic content of the phytopathogen *Xanthomonas axonopodis* pv. *citri*, we found some difficulty in setting up the experiments. Notably, the peak of the internal reference of concentration was specially altered and reduced in size, suggesting that a chemical interference was occurring. The distortion observed in the sample would make any attempts of metabolite identification and/or quantification in the ^1^H-NMR spectrum questionable especially when employing the targeted profiling approach [16]. We attributed this interference to the presence of the polysaccharide constituents of the xanthan gum. The first part of this section describes our efforts to eliminate it, with results for the four methodological interventions tested, and a suggestion of which of them seems to be the most suitable method for the xanthan gum removal. The second part is an extension of the first: once defined a method for gum depletion, decreasing volumes of cells were subjected to the chosen protocol, as a way of defining the trustable threshold of the technique.

### 2.1. Selection of the Best Intervention Procedure for Xanthan Gum Removal

**Figure 1:**
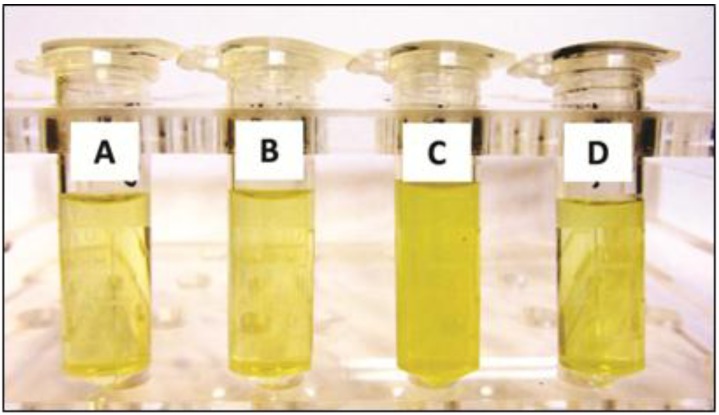
Differences observed in the cellular supernatants of *X. citri* after treatment with four protocols for metabolic extraction. (**A**) three washing cycles; (**B**) five washing cycles; (**C**) conventional centrifugation; and (**D**) ultracentrifugation. The opaque, dark yellow supernatant in c indicates that xanthan gum components remained in the sample.

As no references for the xanthan gum removal in metabolomic studies could be found, we planned four different intervention methods on the basic M/C intracellular content extraction procedure of the *X. citri* metabolome studies. These interventions consisted on the insertion of (i) 3 or (ii) 5 cell washing cycles with a TBS buffer before the cell lysis (by sonication in a M/C solution); (iii) conventional centrifugation at 3.1 × 10^3^ *g* or (iv) ultracentrifugation at 8.0 × 10^4^ *g* step after cell disruption. At the final phase of all tested protocols, cell lysates were centrifuged (in different velocities, depending on the protocol) and the supernatant was collected for solvent drying. Figure 1 illustrates the variation in color and opacity seen in the supernatants obtained from the four different protocols. The method based on conventional centrifugation was not capable of removing xanthan gum components (Figure 1C), as suggested by the opacity of the sample. Conversely, more cycles of washing and ultracentrifugation clearly produced better results.

#### 2.1.1. Cell-Washing Protocols Lead to a Marked Decrease in Overall Intracellular Metabolites’ Retrieval, but One in Particular Followed the Opposite Trend

**Figure 2:**
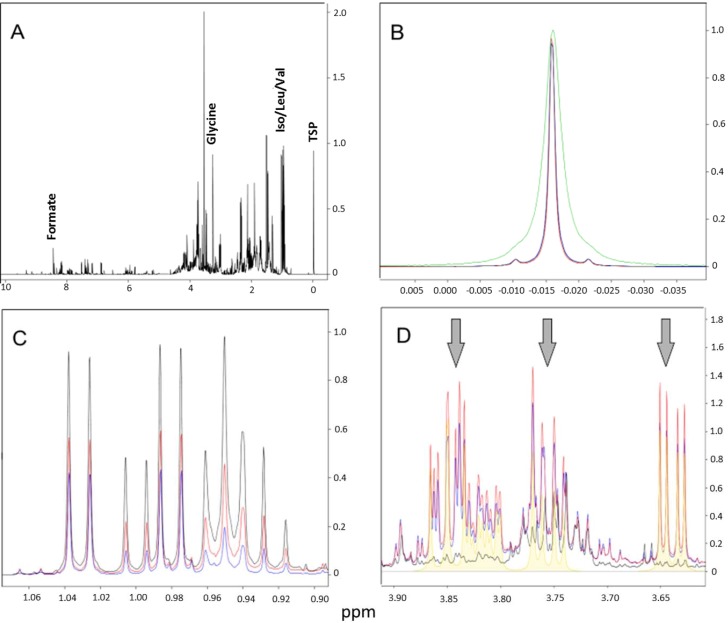
(**A**) Spectra overview of ultracentrifugation protocol; (**B**) TSP signal across samples. Detail for the distorted and broadened peak for the “conventional centrifugation” sample, possibly due to its reaction with xanthan gum constituents vestiges; (**C**) Differential metabolite concentrations across samples. The overall reduced peak intensities in washed samples could be an indicative of metabolic loss and/or cell lysis; (**D**) The intense peaks of trehalose (shaded in yellow under arrows) found in the washed samples. (Black = ultracentrifugation; red = three washing cycles; blue = five washing cycles; green = conventional centrifugation).

The conventional centrifugation sample spectrum allowed a linewidth resolution of around 1.80 Hz for the internal reference of concentration (TSP) after shimming, whereas other sharp singlet peaks along the spectrum exhibited linewidth values around 0.80 Hz (Figure 2b). This finding confirmed the suspicion that gum vestiges remained in the sample, given its turbid aspect (Figure 1).

As expected, spectra from pellets washed 3 or 5 cycles before lysis showed a reduced intensity for the majority of the peaks compared to those from the ultracentrifuged sample. This characteristic was even more pronounced in the 5-washing-cycles sample, suggesting a correlation between cell washes and loss of the metabolic content (Figure 2c). Following the opposite tendency, signals of astonishing intensity appeared in the 5-washing-cycles sample, with decreasing intensities for the 3-washings and the ultracentrifugation sample (Figure 2d). These peaks were identified as belonging to trehalose—a disaccharide implicated in anhydrobiosis (the ability of plants, animals and microorganisms to withstand prolonged periods of desiccation). Table 1 brings the list of the metabolites found in each of the spectra with their respective concentrations.

Spectra analysis and metabolite concentrations indicated that cell-washing protocols indeed eliminated most of the extracellular xanthan gum components; however, at the cost of losing a considerable portion of the internal metabolites possibly due to cell loss and/or the disruption of its membrane. In addition, large amounts of trehalose found in these samples indicated that bacteria suffered considerable hydric stress during the washing process; even though they were performed with isotonic buffer.

Ultracentrifugation presented two major advantages, compared to the cell-washing protocol: (1) it required a minimum of extra intervention, with no addition of solvents or other chemical compounds; (2) the ultracentrifugation step occurred after cell lysis, thus avoiding disturbances in the always-responsive intracellular metabolic content of a living cell. We believe this technique could be extended to other types of organisms, aiming the removal of other matrixes and gums with varied chemical composition; however, we recommend other authors to perform pilot studies on their systems. In combination, all these results indicated that the insertion of an ultracentrifugation step proved to be the most appropriated protocol improvement for the xanthan gum removal for ^1^H-NMR-based metabolomic experiments.

**Table 1:** Metabolites concentrations (µM) identified in the different protocols. The “conventional centrifugation” sample is missing due to impairments in its TSP signal (concentration reference), making any quantification attempt invalid. In bold, the highest value found for every metabolite. Once a protocol presented the highest value for a metabolite, it scored 1 point; at the end of the table, the sum of scores indicated that ultracentrifugation is the method with the best results.

Metabolites	Ultracentrifugation	3 washing cycles	5 washing cycles
**2-Aminoadipate**	**1,659.9**	1,638.5	1,608.5
**2-Methylglutarate**	21.6	**57.7**	44.3
**3-Hydroxybutyrate**	186.2	**992.4**	515.5
**3-Hydroxyisovalerate**	**12.3**	9.0	5.4
**4-Aminohippurate**	**48.1**	20.9	24.7
**AMP**	24.2	**36.9**	29.6
**ATP**	42.7	54.9	**56.9**
**Acetate**	**1,044.2**	941.1	842.0
**Adenine**	**39.2**	35.6	33.1
**Adenosine**	129.1	**305.2**	282.9
**Alanine**	2,817.5	**3,519.6**	1,996.2
**Asparagine**	171.6	**260.3**	153.1
**Aspartate**	805.9	**1,175.2**	997.6
**Beta-Alanine**	**497.8**	358.2	313.6
**Betaine**	741.9	**885.4**	789,0
**Cytidine**	199.1	**247.0**	183.6
**Dimethylamine**	15.4	21.0	**22.4**
**Dimethylformamide**	2.2	**6.9**	N.Q.
**dTTP**	**105.6**	64.7	63.0
**Formate**	1,118.5	1,201.3	**1,387.3**
**Fumarate**	**70.0**	11.3	17.6
**GTP**	51.7	96.2	**119.9**
**Glucose**	902.2	**4,351.7**	3,225.6
**Glutamate**	**9,596.4**	9,021.5	6,871.9
**Glutamine**	**2,134.1**	1,336.5	1,277.5
**Glycerol**	283.6	**408.9**	N.Q.
**Glycine**	**4,289.0**	2,848.7	2,511.9
**Guanosine**	**128.2**	97.8	66.4
**Hypoxanthine**	**718.7**	339.8	287.4
**Inosine**	**200.0**	108.7	92.2
**Isoleucine**	**1,851.1**	841.9	365.1
**Isovalerate**	**61.6**	37.0	11.0
**Lactate**	**159.8**	154.5	125.4
**Leucine**	**3,041.4**	1,492.0	564.5
**Lysine**	**2,699.0**	1386.3	1,019.9
**Methionine**	**878.7**	539.0	282.3
**Methylhistidine**	**68.8**	11.1	0
**NAD+**	**498.2**	476.7	484.3
**NADP+**	60.3	60.7	**66.4**
**Niacinamide**	**84.2**	43.1	28.0
**Phenylalanine**	**1,218.1**	376.4	76.4
**Proline**	1,453.1	**1,693.5**	1,139.6
**Pyroglutamate**	**612.1**	451.2	154.5
**Succinate**	89.0	**897.2**	864.3
**Threonine**	1,204.5	**1,286.8**	663.5
**Thymidine**	**29.2**	23.9	19.5
**Trehalose**	306.6	9,496.0	**11,356.6**
**Trimethylamine**	13.0	12.0	**14.0**
**Tryptophan**	**297.6**	149.1	77.9
**Tyrosine**	**782.2**	496.1	353.6
**UDP-Acetylglucosamine**	**80.2**	63.7	50.6
**UDP-galactose**	**66.2**	54.8	36.8
**UDP-glucose**	**114.0**	75.7	69.3
**UDP-glucuronate**	**25.8**	21.0	20.0
**Uracil**	**673.0**	203.1	233.3
**Uridine**	**224.5**	147.0	108.3
**Urocanate**	0	**4.0**	0
**Valine**	**3,673.5**	2,361.9	1,628.9
**Xanthosine**	4.9	**6.5**	7.8
**SCORE**	**35**	**17**	**7**

N.Q.: non quantifiable.

#### 2.1.2. Decrease in Overall Metabolites’ Concentration in Cell-Washing Protocols is Due to Cell Loss

After comparing the quality of the spectra of the *X. citri* cellular supernatants obtained from four protocol interventions, as well as the values of metabolite concentrations, it was possible to conclude that the “ultracentrifugation” method seemed to be the most appropriate intervention in the M/C extraction protocol for gum removal in *Xanthomonas*. However, the lower metabolite’s concentrations found in both 3- and 5-washed samples compared to the ultracentrifuged one raised the question of whether such reduction would be due to cell loss or simply to bacterial metabolic released during the washing process.

To answer this question, we plated *Xanthomonas* previously submitted to the same washing protocol for gum removal, with aliquots washed for 3 or 5 cycles in TBS 1×. Along eight serial dilutions, colonies were counted for unwashed (control), 3- and 5-times washed bacteria aliquots. The results are shown in Table 2. Considering 1:10 the factor of dilution across the series and the fact that the unwashed aliquot had to be diluted two more times (thus, 100× in absolute scale) than the washed ones for their colonies numbers to reach a countable scale, these experiments indicate that the washing process for xanthan gum removal might be harmful to bacteria, causing cell loss by extrication or death.

**Table 2:** Colony counting on a 1:10 scale serial dilution for 3-, 5-times-washed and unwashed (control) aliquots of *X. citri*. Noteworthy is that the control aliquot had to be diluted two more times than the washed samples for their colonies enter a countable scale, suggesting that bacteria-washing process causes cell loss. ID = indeterminable due to high number of colonies.

Dilution	Unwashed	3 washing cycles	5 washing cycles
**1:10^3^**	ID	ID	ID
**1:10^4^**	ID	800/816	656/680
**1:10^5^**	ID	146/190	126/156
**1:10^6^**	664/672	17/24	30/46
**1:10^7^**	480/696	0/1	6/6

### 2.2. Lower Amounts of Cells for Metabolomic Studies by ^1^H-NMR

Sample size and availability has always been one of the major problems in biological experiments. High costs, waiting time and specimen rarity—or any combination of them—are commonly the principal reasons why big sampling groups are not always ready at the researcher hands. To circumvent this issue and aiming at a thorough study of *X. citri* by metabolic profiling, we searched for the lower limits of *X. citri* cells pellet size for metabolomic studies using ^1^H-NMR spectroscopy, using a 5 mm triple resonance cold probe on a 600 MHz machine (Agilent, Inc., Santa Clara, CA, USA). Four volumes of samples were tested: 500 μL, 250 μL, 125 μL and 62.5 μL.

Differences between pellets began already at the sample preparation stage. The reduced volume of cells in the 62.5 μL vial produced a thin, fragile interphasic proteic ring, between the polar and apolar phases, which did not resist the high pressures of ultracentrifugation. Whereas no occurrences happened with the 125 μL or bigger samples, the interphasic proteic ring that separates the supernatant, methanolic phase, from the apolar, chloroformic phase of the 62.5 μL pellet samples, cracked during ultracentrifugation (data not shown). It is plausible to assume that this crack led, at least to some extent, to the mixture of phases during the supernatant collection step, thus interfering with the internal reference integrity (as observed by the great difficulty in shimming the sample) and compromising metabolites’ quantification (data not shown). Table 3 brings the metabolite’s concentrations found in the three available pellet volumes.

**Table 3:** Concentration of metabolites (μM) identified in the 500 μL, 250 μL and 125 μL pellets submitted to ultracentrifugation prior to the analysis. (Mean ± SD, *n* = 3 for each group).

	Pellet volumes		
**Metabolites**	**500 μL**	**250 μL**	**125 μL**
**2-Aminoadipate**	653.9 ± 12.6	305.9 ± 33.2	148.6 ± 13.1
**2-Methylglutarate**	55.9 ± 2.5	21.9 ± 7.5	7.0 ± 0.2
**3-Hydroxybutyrate**	235.5 ± 22.7	115.2 ± 12.9	52.4 ± 4.4
**3-Hydroxyisovalerate**	116.8 ± 18.2	73.3 ± 5.9	30.4 ± 5.0
**4-Aminohippurate**	84.6 ± 4.9	30.2 ± 9.1	11.9 ± 1.2
**AMP**	1.9 ± 0.4	7.1 ± 4.3	12.7 ± 4.0
**ATP**	75.3 ± 3.5	32.1 ± 5.7	17.5 ± 3.3
**Acetate**	914.3 ± 173.7	470.7 ± 116.8	213.8 ± 26.7
**Adenine**	124.7 ± 8.5	58.0 ± 13.3	29.4 ± 4.4
**Adenosine**	268.6 ± 49.5	121.8 ± 20.8	49.6 ± 5.1
**Alanine**	4,475.6 ± 226.7	1,999.3 ± 383.6	1,042.9 ± 89.6
**Asparagine**	188.2 ± 7	90.3 ± 22.6	51.2 ± 8.1
**Aspartate**	1,579.5 ± 145.9	738.3 ± 100.8	368.0 ± 34.6
**Beta-Alanine**	288.3 ± 30.4	166.3 ± 16.1	79.3 ± 4.6
**Betaine**	842.9 ± 31.0	377.6 ± 52.3	188.7 ± 12.4
**Cytidine**	196.8 ± 19.7	54.4 ± 18.3	25.8 ± 2.1
**Dimethylamine**	3.2 ± 0.8	2.3 ± 0.2	1.0 ± 0.1
**Dimethylformamide**	26.1 ± 4.5	11.5 ± 2.2	4.4 ± 1.4
**dTTP**	145.5 ± 2.5	77.3 ± 8.6	41.0 ± 3.8
**Formate**	1,517.6 ± 250.1	801.4 ± 130.0	478.1 ± 40.3
**Fumarate**	9.4 ± 1.8	4.2 ± 0.7	2.2 ± 0.2
**GTP**	53.6 ± 2.9	21.9 ± 7.9	12.4 ± 2.9
**Glucose**	740.3 ± 51.6	279.8 ± 63.2	149.0 ± 18.4
**Glutamate**	12,186.7 ± 401.5	5,571.3 ± 595.8	2,725.4 ± 244.0
**Glutamine**	2,820.6 ± 15.9	1,411.0 ± 218.3	694.5 ± 58.8
**Glycerol**	781.2 ± 41.5	332.6 ± 34.2	210.9 ± 46.5
**Glycine**	5,078.8 ± 401.2	2,324.3 ± 371.9	1,162.5 ± 97.6
**Guanosine**	132.7 ± 14	55.2 ± 16.1	26.6 ± 2.4
**Hypoxanthine**	877.9 ± 18.8	376.6 ± 69.3	167.1 ± 15.4
**Inosine**	161.6 ± 35.2	33.3 ± 8.4	19.6 ± 3.2
**Isoleucine**	2,668.0 ± 105.8	1,259.1 ± 182.3	654.6 ± 63.3
**Isovalerate**	255.3 ± 27.2	121.5 ± 13.6	45.2 ± 3.0
**Lactate**	279 ± 38.7	123.3 ± 18.6	66.7 ± 12.1
**Leucine**	4,479.2 ± 107.7	2,059.9 ± 343.0	1,084.8 ± 104.2
**Lysine**	3,142.5 ± 250.2	1,630.0 ± 138.5	898.5 ± 43.6
**Methionine**	1,243.4 ± 56.3	614.5 ± 80.6	335.8 ± 21.4
**Methylhistidine**	127.2 ± 8.9	50.0 ± 7.0	23.6 ± 1.5
**NAD+**	499.1 ± 33.1	234.8 ± 25.1	110.4 ± 10.3
**NADP+**	40.6 ± 3.2	15.7 ± 5.8	9.5 ± 0.4
**Niacinamide**	170.5 ± 16.4	78.9 ± 7.5	33.8 ± 4.2
**Nicotinate**	73.6 ± 6.2	32 ± 3.8	14.8 ± 1.0
**Nicotinic acid adenine dinucleotide**	18.3 ± 2.5	8.7 ± 1.3	4.9 ± 1.6
**Phenylalanine**	2,213.1 ± 39.9	1,062.5 ± 123.9	544.5 ± 33.0
**Proline**	2,132.1 ± 145	843.2 ± 192.5	453.3 ± 57.4
**Pyroglutamate**	1,360.7 ± 109.1	629.0 ± 88.0	285.6 ± 28.2
**Succinate**	423.9 ± 36	147.4 ± 37.2	71.0 ± 9.8
**Threonine**	2,494.9 ± 149.1	1,120.0 ± 150.2	564.9 ± 44.7
**Thymidine**	35.4 ± 1.4	9.2 ± 4.3	3.9 ± 0.2
**Trehalose**	313.8 ± 39.4	182.3 ± 27.1	73.1 ± 5.5
**Trimethylamine**	9.9 ± 0.9	7.6 ± 0.9	6.4 ± 0.9
**Tryptophan**	432.3 ± 30.4	244.2 ± 44.4	140.0 ± 14.8
**Tyrosine**	1,266.3 ± 48.1	626.3 ± 80.2	311.1 ± 26.8
**UDP-Acetylglucosamine**	66.7 ± 4.2	28.6 ± 4.2	18.1 ± 0.6
**UDP-galactose**	68.3 ± 2	32.1 ± 6.3	16.9 ± 1.1
**UDP-glucose**	147.8 ± 7	78.8 ± 10.8	43.1 ± 3.1
**UDP-glucuronate**	22.0 ± 0	9.4 ± 1.0	5.8 ± 1.3
**Uracil**	1,013.9 ± 101.6	438.5 ± 57.4	199.4 ± 14.0
**Uridine**	198.4 ± 15	61.7 ± 17.0	22.6 ± 0.5
**Urocanate**	16.0 ± 2.1	9.1 ± 1.6	3.5 ± 0.4
**Valine**	4,583.7 ± 167.1	2,167.1 ± 296.2	1,106.0 ± 84.9
**Xanthosine**	19.5 ± 0.5	8.5 ± 1.2	4.2 ± 0.2

**Figure 3:**
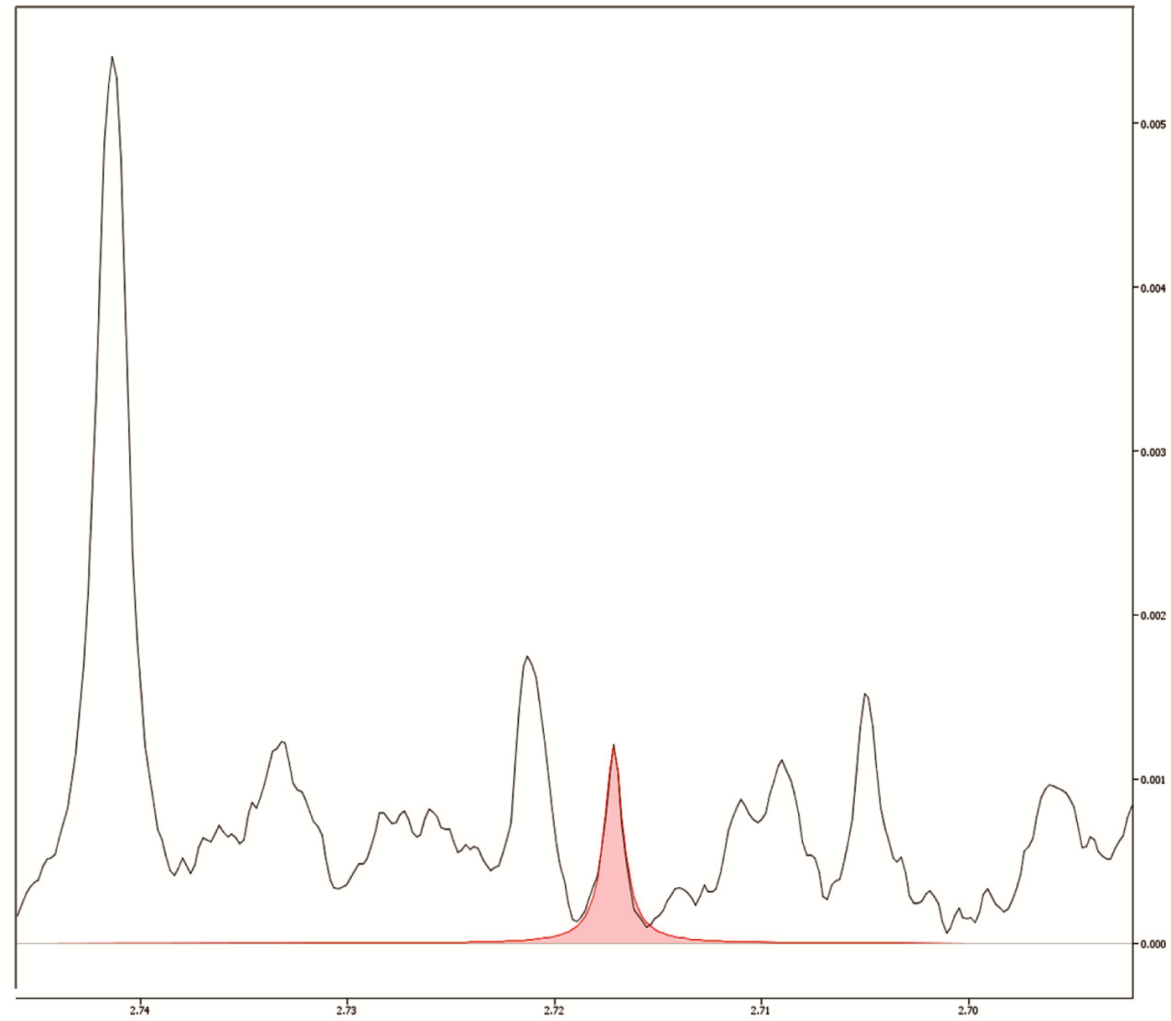
Detail of the 125 µL pellet spectrum. In red, the fitting of the dimethylamine peak, whose concentration was the lowest among all the metabolites detected. As occurred to all compounds, its marked, well-defined peak provided a high signal:noise ratio and, therefore, a reliable quantification.

These results suggest that pellets as small as 125 µL could be used in a ^1^H-NMR-based metabolomic investigation without losses in metabolic detection, in a similar NMR setup. Considering our metabolite detection limit, based on the Chenomx peak identification protocol, we found no metabolite that reached undetectable levels as the pellet volumes decreased, suggesting that pellets with 125 µL could be as informative as pellets four times bigger (Figure 3). Indeed, we believe that even smaller pellets could still be successfully employed in metabolomics studies if centrifuge tubes with reduced diameters were used, thus yielding thicker inter-phase protein rings and ensuring sample’s integrity during the ultracentrifugation process. Our research group is starting to explore low speed/low volume Magic Angle Spinning (MAS) in high-resolution solid-state NMR (HR-MAS-NMR) methods, which may enable studies with small pellet and lower volume samples obtained from extraction procedures.

## 3. Experimental Section

### 3.1. Bacterial Strains and Cell Culture Growth Conditions

Cultures of *Xanthomonas axonopodis* pv. *citri* 306 were prepared by inoculating a single colony in 500 mL of Luria Bertani Broth medium (LB), containing tryptone (10 g), yeast extract (5 g) and NaCl (10 g) per liter of water [17]. Bacteria were left to grow overnight at 200 rpm shaking and 30 °C and harvested at 1.0 × 10^4^ *g* for 15 min. Cell proliferation until stationary phase was monitored by OD_600_, when pellets proceeded for metabolites recovery.

### 3.2. Metabolic Extraction Procedures

The here named “M/C protocol without specific adaptations” for metabolic recovery was adapted from Le Belle *et al.* [15]. A major adaptation was an increase in the solvent volumes due to the large volume of cell pellets studied. This extraction method consisted in adding to the pellets methanol and chloroform (4 °C) in a ratio of 2:1 (v/v, 2.5 mL/bacteria pellet). The cell pellet-solvent mixtures, immersed in iced water, were sonicated for 3 minutes with a 10 s interval between each minute. Chloroform and distilled water (4 °C) were then added to the samples in a ratio of 1:1 (2.5 mL/bacteria pellet). After a brief vortexing to form an emulsion, the samples were centrifuged at 3.116 × 10^3^ *g* for 20 min at 4 °C. The upper phase (methanol, water and polar metabolites) was collected and totally dried in a vacuum concentrator (Speedvac) at low speed and without heating.

For xanthan gum removal, the tested adaptations here called “3- and 5-washing-cycles” consisted of washing the bacteria pellets in 10 mL of TBS 1× buffer, with subsequent cell recovery at 1.152 × 10^4^ *g* for 10 min between each wash. This step occurred before the metabolic extraction procedure detailed above.

The “conventional centrifugation” and “ultracentifugation” procedures were very similar to the “M/C protocol without specific adaptations”, with the exception that samples were centrifuged at 1.555 × 10^4^ *g* and 8.0 × 10^4^ *g*, respectively*.* In both cases, time and temperature of centrifugation were kept the same as the original protocol.

### 3.3. Colony Formation Units

Successive 10-fold serial dilutions were done with bacterial pellets submitted to none, 3 and 5 washing cycles for colony forming units monitoring. Pellets were diluted in TBS 1× until 1:10^−7^ and plated on 90 × 15 mm polystyrene dishes containing 25 mL of LB medium supplemented with ampicillin (100 mg/mL). Serial dilutions were done twice from each pellet. The bacterial colonies were counted after 48 h of incubation at 28 °C.

### 3.4. Sample Preparation for NMR Analysis

After drying the samples in a vacuum concentrator (Speedvac), the remaining solid phase was rehydrated in 600 µL of deuterium oxide (D_2_O, 99.9%; Cambridge Isotope Laboratories Inc., Tewksbury, MA, USA), containing phosphate buffer (0.1 M, pH 7.4) and 0.5 mM of TSP (3-(Trimethylsilyl)propanoic acid; Sigma-Aldrich) for internal reference. Samples were added to a 5 mm NMR tube for immediate acquisition.

### 3.5. NMR Data Acquisition and Metabolite Identification

^1^H-NMR spectra of samples were acquired using a Agilent Inova NMR spectrometer (Agilent Technologies Inc., Santa Clara, CA, USA) equipped with a 5 mm triple resonance cold probe and operating at a ^1^H resonance frequency of 600 MHz and constant temperature of 298 K (25 °C). A total of 128 free induction decays (FIDs) were collected with 32 K data points over a spectral width of 16 ppm. A 1.5 s relaxation delay was incorporated between FIDs, during which a continual water pre-saturation radio frequency (RF) field was applied. Spectral phase and baseline corrections, as well as the identification and quantification of the metabolites present in the samples, were performed using the Chenomx NMR Suite 7.6 software (Chenomx Inc., Edmonton, AB, Canada).

## 4. Conclusions

An appropriate pre-treatment step is essential for an efficient removal of the xanthan gum from *Xanthomonas*’ pellets prior to NMR spectroscopy experiments. The ultracentrifugation intervention presented the best results in terms of both spectra quality and potential for metabolite quantification. Conventional cell-washing protocols exhibited an overall decrease in metabolite’s concentrations due to cell extrication or death along the washing cycles. In addition to its ease of implementation, a minimal extra manipulation after cell lysis—thus avoiding disturbances in the metabolic milieu of the living system—can be pointed as an advantage of the ultracentrifugation intervention for the xanthan gum removal. We don’t claim to have exhausted all the alternatives to solve this issue, but to start answering questions we faced during answering our own research questions, which may be of interest to others, and as an exercise in collaboration and creativity in the solving of new problems. 

## Supplementary Files

Supplementary File 1 Supplementary (XLSX, 29 KB)

## Abbreviations

D_2_O: deuterium oxide
FID: free induction decay
^1^H-NMR: proton nuclear magnetic resonance
HR-MAS-NMR: high resolution solid-state NMR
LB: Luria Bertani Broth medium
MAS: magic angle spinning
M/C: methanol/chloroform
OD: optical density
RF: radio frequency
TBS: tris-buffered saline
TSP: 3-(Trimethylsilyl)propanoic acid
*X. citri*: *Xanthomonas* *axonopodis* pv. *citri* 306
